# Antidepressant use pattern and disparities among cancer patients in the United States

**DOI:** 10.3389/fpubh.2022.1000000

**Published:** 2022-11-09

**Authors:** Jingrui Zou, Yong Zhu

**Affiliations:** ^1^Department of Scientific Affairs, Tongji Hospital, Tongji Medical College, Huazhong University of Science and Technology, Wuhan, China; ^2^Wayzek Science, St. Paul, MN, United States

**Keywords:** antidepressant, cancer, depression, epidemiology, health disparities

## Abstract

Many cancer patients also suffer from depression, however, pharmacotherapy of depression and related disparities in US cancer survivors have not been examined in a nationally representative sample. In the present study, 2,590 adult cancer survivors participating in the National Health and Nutrition Examination Survey 2011–2020 were included and antidepressant use pattern was investigated. To examine disparities by social-demographic characteristics and access to healthcare, multivariate logistic regression analysis was conducted in 422 cancer patients who were using antidepressants and 230 cancer patients who were not using antidepressants but were diagnosed with depression. Results suggested that 21% of adult cancer survivors were using antidepressants and selective serotonin reuptake inhibitors were the most common type of antidepressants used. Antidepressant users were more likely to be female, non-Hispanic white, those who were married or living with partner. In addition, those without a routine place to go for healthcare were less likely to use antidepressants. Disparities were not found by age, family income levels, education, or health insurance coverage. The findings highlight disparities in antidepressant use in cancer patients in the US. Policy makers need to better allocate healthcare resources and facilitate availabilities of affordable care to every patient in need.

## Introduction

Depression in cancer patients is a common mental health condition. It was reported that 16.5% of cancer patients in palliative care settings were diagnosed with depression (1). While prevalence of depression varies by the type and stage of cancer, it negatively affects cancer patients' quality of life (2) and long-term survival outcome (3, 4). It also increases healthcare cost and utilization (5). For example, it was estimated US Medicare beneficiaries with cancer who had newly diagnosed depression had about 47% higher monthly medical cost than cancer patients without depression (6).

Antidepressants have been prescribed for treatment of depression in cancer patients. Despite the need in disparity research for depression in cancer patients (7), very few nationally representative studies have examined disparities in antidepressant use in this population. A previous study indicated that 18.3% of adult cancer survivors used antidepressants in 1999–2012 in the US, however the study did not report whether the use rate varied by social-demographic characteristics of cancer patients (8). Another national study found that US adult cancer patients were more likely to report medication use for depression in 2010–2013, compared to adults with no history of cancer; also, there was a disparity such that female patients, non-Hispanic white, and those widowed or divorced were more likely to use antidepressants (9). Because the comparison was made to the general public without cancer diagnosis, it is not known whether the observed disparity in antidepressant use was true or was a reflection of differences in prevalence of depression in cancer patients, as certain groups of cancer patients were more likely to have depressive symptoms (10) and this may have accounted for a higher likelihood of antidepressant use in these groups.

The objective of the study was to examine antidepressant use patterns among adult cancer survivors using recent US nationally representative data with a focus on potential disparities due to social-demographic characteristics or access to healthcare. Information on this topic will fill the gap in the literature and inform healthcare policy and facilitate optimal allocation of medical resources that may help eliminate inequality in healthcare at the national level.

## Methods

### Data source

The National Health and Nutrition Examination Survey (NHANES) is a research program conducted by the US National Center for Health Statistics (NCHS) to assess health and nutrition status of US residents (11). The continuous NHANES program involves a complex multi-stage sample design, and the research team conducts a household interview and a medical examination at a mobile examination center in about 5,000 participants each year, as a nationally representative sample, with data released in 2-year cycles since 1999, and the data are publicly available (11). For the 2019-2020 survey cycle, data collection was suspended in March 2020 because of the COVID-19 pandemic; in order to keep the nationally representative nature of the data, the NCHS combined the NHANES 2019-March 2020 data with NHANES 2017-2018 data, readjusted the sample weight and released them as the NHANES 2017-March 2020 Pre-pandemic data (12). To provide accurate estimate on a subgroup that may not be very common in the US general population, it was recommended to combine NHANES data from adjacent survey cycles in order to increase sample size (13). In the present study, data from NHANES 2011–2012, 2013–2014, 2015–2016, 2017-March 2020 were pooled (for 9.2 years of data, referred as NHANES 2011–2020 thereafter). The NCHS Ethics Review Board approved the NHANES protocol and each adult participant provided their consent to participate in the study.

### Antidepressant use

The NHANES team collected information on prescription medication taken in the past 30 days as reported by participants during the household interview, and whenever possible, the interviewer examined the medication containers of products used. Each medication was classified using a 3-level nested therapeutic classification system using Lexicon Plus, a database containing information of all prescription drug products in the US (14). Antidepressants were identified by the second level code of 249 (8). Classification of antidepressants were based on the third level codes. A full list of antidepressants and their classifications can be found in Supplementary Table 1.

### Social-demographic characteristics

Age, gender, race/ethnicity, education, marital status, and ratio of family income to poverty were obtained from the NHANES demographic data, and recoded as categorical variables as needed. For example, the ratio of family income to poverty were classified as low income (≤1.85), middle income (1.86–3.49), and high income (≥3.50), based on income-eligibility for federal food assistance programs, accounting for the number of individuals in the household (15).

### Access to healthcare

Whether a participant is covered by a health insurance plan was inquired during the household interview. In addition, information about whether there is a place they usually go when they are sick or need advice about health was collected during the household interview. Both responses were coded as Yes or No according to answers to the interview questions.

### Depression assessment

Participants completed the PHQ-9 questionnaire, a validated instrument for depression diagnosis and severity measure (16, 17) at the mobile examination center. The survey consisted nine questions related to frequency of depression symptoms in the past 2 weeks. PHQ-9 has been validated for use in cancer patients; a cut-off score of 8 points could provide a sensitivity of 93% and a specificity of 81% to identify major depressive disorders in cancer patients (18). Participants who skipped one of the nine questions were included with the missing score of the question replaced by the average score of the other eight questions (19). A patient was considered to be depressed if the total score of PHQ-9 is eight points or higher.

### Study population

To examine antidepressant use pattern, the study population included 2,590 adults aged 20 years or older who completed the NHANES interview and responded “yes” to the question “have you ever been told by a doctor or other health professional that you had cancer or a malignancy of any kind?”

To examine disparities in antidepressant use, the study included 422 cancer survivors who reported antidepressant use and completed a depression assessment at the mobile examination center, and the study then compared these patients with 230 cancer survivors who did not use antidepressants but were diagnosed with depression from the PHQ-9 questionnaire.

### Statistical analysis

SAS 9.4 (SAS Institute, Cary, NC, USA) was used for statistical analysis. 9.2 years of sample weight was applied with SAS Survey procedures being used to account for the complex multi-stage NHANES study design (13). Prevalence of antidepressant use in cancer survivors was estimated by NHANES data cycles, followed by a descriptive analysis by the class of antidepressants. To assess potential disparity in antidepressant use due to social-demographic characteristics or access to healthcare, analysis was restricted to a group of participants who also completed the depression assessment, with a comparison between antidepressant users and those who did not use antidepressants but were diagnosed with depression, using multivariate logistic regression for survey data with all variables related to social-demographic characteristics and access to healthcare included in the model. Frequency data were presented as n (weighted n, weighted percentage). Results from logistic regression were presented as adjusted odds ratio (aOR) with their 99% confidence intervals (CI) to account for possible multiplicity issues in the model.

## Results

### Antidepressant use pattern

**Figure 1:**
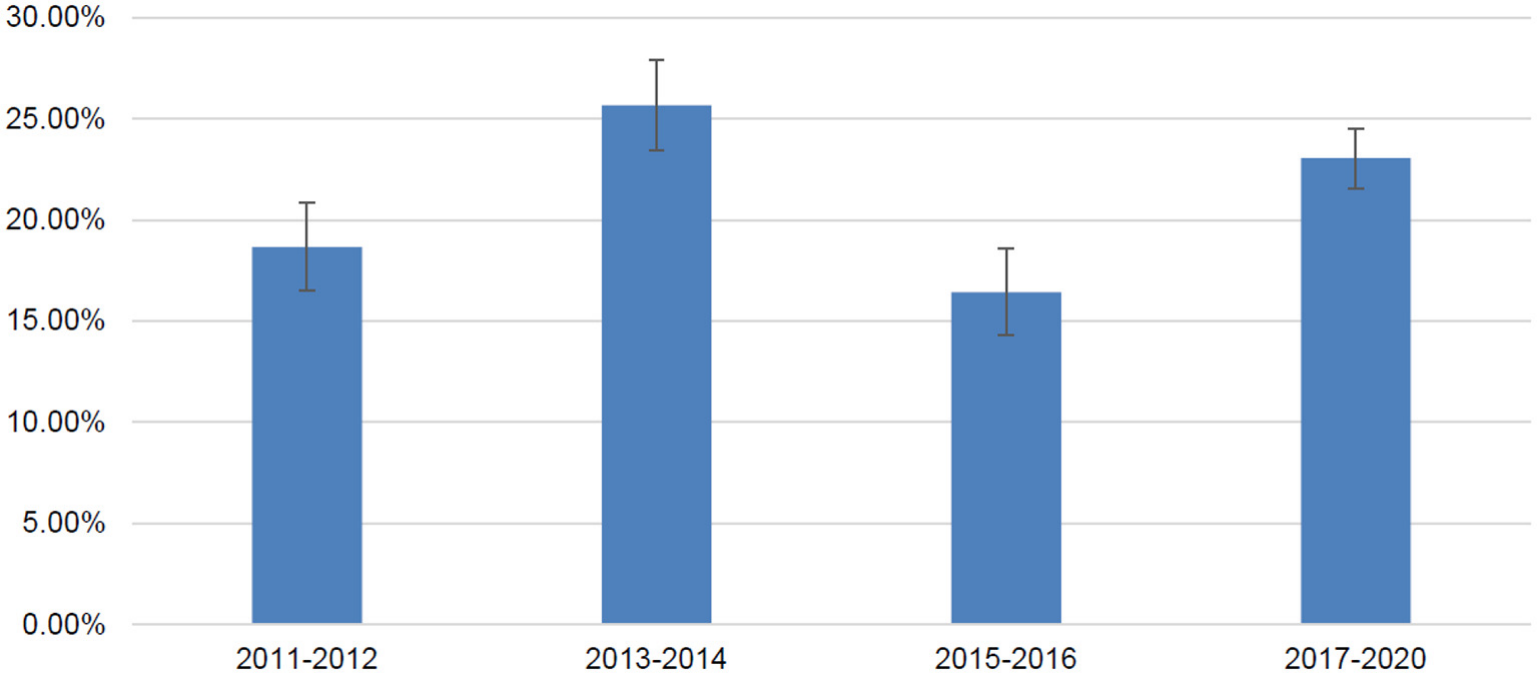
Prevalence of antidepressant use in adult cancer survivors in United States by NHANES data cycles.

Among 2,590 adult cancer survivors from NHANES 2011-2020, 491 patients reported antidepressant use; the weighted number of adult cancer survivors was 25594234 and the weighted number of antidepressant users were 5460460. The overall prevalence of antidepressant use among adult cancer survivors was 21.33% (standard error 0.98%). Prevalence of antidepressant use in difference NHANES data cycles is presented in Figure 1. The prevalence varied between 16.43% (from NHANES 2015–2016) and 25.67% (from NHANES 2013–2014) without a significant linear trend over the time period examined in the study. When data were pooled together, selective serotonin reuptake inhibitors were the most common type of antidepressants used in this population, followed by serotonin and norepinephrine reupdate inhibitors, phenylpiperazine, tricyclic antidepressants, tetracyclic antidepressants, and monoamine oxidase inhibitors (Table 1).

**Table 1:** The type of antidepressant medications used by adult cancer survivors, NHANES 2011–2020.

**Antidepressant drug class**	**Number of users**	**Weighted number of users**	**Percent in antidepressant users (standard error)**
Selective serotonin reuptake inhibitors	283	30,74,202	56.30% (2.78%)
Serotonin and norepinephrine reupdate inhibitors	88	11,34,279	20.77% (2.74%)
Phenylpiperazines	67	7,87,278	14.42% (1.83%)
Tricyclic antidepressants	63	6,07,845	11.13% (1.69%)
Tetracyclic antidepressants	24	2,44,579	4.48% (1.00%)
Monoamine oxidase inhibitors	2	19,027	0.35% (0.31%)
Miscellaneous	46	6,20,054	11.35% (2.25%)

### Social-demographic characteristics and access to healthcare by antidepressant use

Among adult cancer survivors who completed a depression assessment; 422 (weighted sample size 5212240) used antidepressants and 230 (weighted sample size 1819723) had depression but did not use any antidepressants. Comparison of social-demographic characteristics and access to healthcare between these two groups is presented in Table 2. Age, gender, race/ethnicity, marital status, education, and having a routine place to go for healthcare are significantly associated with antidepressant use in cancer survivors (all *P* < 0.05). Specifically, younger patients, male patients, racial minority groups, those widowed or divorced or separated, those with high school diploma or lower education, and those without a routine place to go for healthcare are less likely to use antidepressants even though they were diagnosed with depression.

**Table 2:** Social-demographic characteristics and access to healthcare by antidepressant use in adult cancer survivors in the United States.

	**Those who used antidepressants (*N* = 422) Weighted *N* = 52,12,240**	**Those with depression diagnosis but without antidepressant use (*N* = 230) Weighted *N* = 18,19,723**	**Chi-square test *P*-value**
Age (years)			0.031
20–40	22 (2,35,170, 4.5%)	18 (2,14,337, 11.8%)	
40–65	170 (25,45,749, 48.8%)	101 (8,46,278, 46.5%)	
65–79	153 (17,54,435, 33.7%)	80 (5,57,990, 30.7%)	
≥80	77 (6,76,886, 13.0%)	31 (2,01,118, 11.1%)	
Gender			0.038
Male	132 (15,46,934, 29.7%)	92 (7,39,787, 40.7%)	
Female	290 (36,65,306, 70.3%)	138 (10,79,936, 59.3%)	
Race/Ethnicity			<0.001
Non-hispanic white	311 (46,32,167, 88.9%)	104 (12,64,167, 69.5%)	
Non-hispanic black	42 (1,56,989, 3.0%)	60 (2,12,783, 11.7%)	
Hispanics	42 (1,55,023, 3.0%)	40 (1,66,536, 9.2%)	
Asian	6 (24,490, 0.5%)	15 (65,185, 3.6%)	
Other, including multi-racial	21 (2,43,571, 4.7%)	11 (1,11,052, 6.1%)	
Ratio of family income to poverty			0.100
≤ 1.85	170 (14,40,468, 30.4%)	124 (7,11,798, 42.4%)	
1.86–3.49	107 (14,12,380, 29.8%)	46 (4,80,294, 28.6%)	
≥3.50	104 (18,887,56, 39.8%)	40 (4,88,654, 29.1%)	
Marital status			0.034
Married/living with partner	219 (32,03,316, 61.5%)	100 (8,25,122, 45.6%)	
Widowed/divorced/separated	166 (15,97,973, 30.7%)	100 (7,99,414, 44.2%)	
Never married	37 (4,10,951, 7.9%)	29 (1,86,115, 10.3%)	
Highest education			0.021
High school diploma or lower	158 (16,38,422, 31.4%)	119 (8,36,949, 46.1%)	
Associate degree	165 (19,50,088, 37.4%)	71 (5,82,490, 32.1%)	
College degree or higher	99 (16,23,729, 31.2%)	39 (3,97,892, 21.9%)	
Covered by health insurance			0.059
Yes	408 (49,74,857, 95.4%)	205 (16,00,731, 89.0%)	
No	14 (23,73,83, 4.6%)	23 (1,97,093, 11.0%)	
Routine place to go for healthcare			<0.001
Yes	411 (50,54,483, 97.0%)	206 (15,08,722, 82.9%)	
No	11 (1,57,757, 3.0%)	24 (3,11,001, 17.1%)	

Data were presented as sample size (weighted sample size, weighted percentage). Counts for certain variables may not add to total number due to missing values.

### Disparities in antidepressant use

Table 3 presents results from multivariate logistic regression analysis for associations between use of antidepressants and social-demographic characteristics and access to healthcare. Results revealed that social-demographic characteristics such as gender, race/ethnicity, and marital status were significantly associated with antidepressant use. For example, female patients were more likely to use antidepressants than male patients (aOR = 3.45, 99% CI = [1.43, 8.32]). Compared to non-Hispanic white patients, various other race/ethnic groups, including non-Hispanic black, Hispanics, and Asian, were 76–96% less likely to use antidepressants. Also, patients who were widowed or divorced or separated were less likely to use antidepressants than patients who were married or living with partner (aOR = 0.39, 99% CI = [0.17, 0.91]). For access to healthcare, those without a routine place to go for healthcare were less likely to use antidepressants despite being depressed (aOR = 0.13, 99% CI = [0.03, 0.50]), respectively. No significant association was found for age, income level, education, or health insurance coverage in the multivariate logistic regression analysis.

**Table 3:** Multivariate logistic regression of antidepressant use (yes vs. no but with depression diagnosis) in adult cancer survivors in the United States.

	**Adjusted odds ratio with 99% confidence interval**
**Age (years)**	
20–40	Reference
40–65	2.45 (0.72, 8.32)
65–79	2.14 (0.61, 7.47)
≥80	2.47 (0.52, 11.72)
**Gender**	
Male	Reference
Female	3.45 (1.43, 8.32)
**Race/Ethnicity**	
Non-Hispanic White	Reference
Non-Hispanic Black	0.19 (0.09, 0.43)
Hispanics	0.24 (0.09, 0.60)
Asian	0.04 (0.01, 0.21)
Other, including multi-racial	0.68 (0.21, 2.15)
**Ratio of family income to poverty**	
≤1.85	Reference
1.86–3.49	0.99 (0.42, 2.34)
≥3.50	1.23 (0.52, 2.87)
**Marital status**	
Married/living with partner	Reference
Widowed/divorced/separated	0.39 (0.17, 0.91)
Never married	0.76 (0.23, 2.53)
**Highest education**	
High school diploma or lower	Reference
Associate degree	1.64 (0.94, 2.87)
College degree or higher	1.65 (0.64, 4.24)
**Covered by health insurance**	
Yes	Reference
No	0.36 (0.07, 1.77)
**Routine place to go for healthcare**	
Yes	Reference
No	0.13 (0.03, 0.50)

All of these variables were included in the multivariate logistic regression model.

## Discussion

Results from the present study indicated that ~1 in every 5 cancer patients were using antidepressants in 2011–2020. Disparities in antidepressant use by social-demographic characteristics and access to healthcare were found; for example, among these with depression, racial minority cancer patients were less likely to use antidepressants than non-Hispanic white patients; those without a routine place to go for healthcare were also less likely to use antidepressants.

The prevalence of antidepressant use in US cancer patients was slightly increased in the past decade, compared to 18.3% in 1999–2012 as reported previously (8). The prevalence is also higher than that in the general US population (20, 21), which is likely due to a higher rate of depression in cancer patients. About 8% of US adults reported depressive symptoms (22) whereas depression could be much more common in cancer patients. For example, a review on prevalence of depression in cancer patients reported a median prevalence ranging from 15 to 29% (23). The prevalence of depression defined by PHQ-9 in this pooled study population was found to be 14.2%. When examined by NHANES data cycles, the prevalence varied from 10.7% (from NHANES 2015–2016) to 16.1% (from NHANES 2013–2014) in the past decade (Supplementary Figure 1). This indicates the prevalence of depression in cancer patients in the present study was at the lower end of the spectrum when compared to data from the published review (23). This is likely because PHQ-9 only accessed depressive symptoms over the past 2 weeks, leading to an underestimate of the prevalence.

While most previous studies on disparities of antidepressant use in cancer patients did not report the type of antidepressants used, the present study also found selective serotonin reuptake inhibitors are the most common type of antidepressants used by cancer patients, which is similar to findings from a previous systematic review on prescribing patterns for cancer patients globally (24) as well as results from patients with depressive disorders in the US (25). With regards to efficacy of antidepressant use in cancer patients, a previous review summarized preliminary evidence from a limited number of randomized controlled trials and suggests they may be able to alleviate depression symptoms (26), however, a recent systematic review concluded a very low certainty evidence on benefits due to the lack of high-quality clinical trials (27). Future studies addressing this urgent need are warranted.

Race/ethnicity was found to be a major predictor among demographic characteristics for antidepressant use in US cancer patients. This is similar to results from previous studies with local or non-nationally representative samples. For example, Parikh et al. found African American patients with prostate cancer who developed depression after cancer diagnosis were less likely to receive antidepressant prescription than White patients (4); similarly, elderly African American patients (28), or other minorities in race (29) were less likely to be prescribed with antidepressants than elderly white patients. This phenomenon of lower antidepressant use in these racial minority groups requires attentions from medical practitioners and healthcare policy makers because studies have shown a higher prevalence of depression following cancer diagnosis in these racial minority groups (4, 30).

Consistent with results in the general adult population (21), female patients were more likely to use antidepressants in the present study. It was also found those who were widowed or divorced were less likely to use antidepressants, similar to a previous study that compared cancer patients with general adult population (9). Nonetheless, income levels were not associated with antidepressant use from adjusted logistic regression analysis. Previous studies have shown a higher prevalence of depression in cancer patients from lower income families (31, 32); the lack of significant association with income may be partly explained by availabilities of federal insurance coverage such as Medicaid to assist with healthcare cost for families with lower income, or due to unmeasured confounding factors. Analysis of antidepressant cost in the US in 2014–2015 revealed that majority of antidepressants were available in a generic form in the US market with an average cost per generic prescription of $57 (33), which was relatively affordable with insurance coverage. Nonetheless, due to lack of information, it was not known whether there was a significant association between antidepressant use with the type of insurance; although comparison between cancer patients and adults without cancer diagnosis in a previous study revealed those with public insurance were more likely to take antidepressants than those with private insurance (9). Lacking a routine place to go for healthcare is associated with less likelihood of antidepressant use in the study. Despite governmental effort for increased health insurance coverage in the United States, the study revealed that 17% of patients who had depression but not treated with antidepressants did not have a routine place to go for healthcare. This highlights the need for better implementation of healthcare policy to eliminate the disparities in access to healthcare at the national level. While the impact on antidepressant use rate or the prevalence of depression is yet to be examined when such policies are implemented, effort should also be made to address known barriers such as uncertainty about treatment and cost (34) for increased adoption of treatment strategies in managing mental health problems in cancer patients.

There are several limitations in the study. First, PHQ-9 was used to define depression in the study population. While it is a valid screening tool for depression in cancer patients, it only measured self-reported symptoms over the past 2 weeks, and there was no baseline diagnostic data prior to antidepressant prescription available in the NHANES data. There were patients who were using antidepressants but not depressed according to results from PHQ-9 at the time of the study. This is likely because these patients may have started the medication due to an earlier diagnosis and after taking the medication for a period of time, their depressive symptoms may have been alleviated, leading to a lower score in PHQ-9 at the time of NHANES data collection. Comparisons of characteristics of antidepressant users by PHQ-9 scores were presented in Supplementary Table 2; those with a PHQ-9 score <8 were more likely to be younger, from low-income families, widowed or divorced or separated, and less likely to have a college or higher degree or health insurance coverage. Second, medication was self-reported in NHANES with medication container being verified by the interviewer when possible; the data was still subject to potential under-reporting. However, data on medication use for depression in NHANES has been validated and allows investigation of antidepressant use pattern at the national level (35). Third, not all patients with depression need to be treated with prescriptive antidepressants; information on over-the-counter medications and psychotherapy management for depression were not available. The prevalence of antidepressant use may be an underestimate for prevalence of treatment for depression in cancer patients. Fourth, as a cross-sectional survey, the NHANES does not conduct any follow up studies for health outcomes; as such, the effect of antidepressant use on quality of life or symptom alleviation in this population was unknown. Fifth, the NHANES 2019–2020 did not include any data during the COVID-19 pandemic; it would be worth investigating how the pandemic may have affected mental health conditions of cancer patients and their access to healthcare, and whether the observed disparities were increased as a result from the pandemic. Despite these limitations, the study was the first study to our knowledge that examined disparities in antidepressant use in US cancer survivors using a nationally representative survey data; its results could be very relevant and helpful from a public health perspective for resources allocation and healthcare policy reforms. The findings highlight the critical need to address existing disparities in managing mental health problems in cancer patients and to eliminate inequality on access to healthcare in the US, particularly in racial minority groups. The study also highlights the increasing need on awareness of mental health problems in cancer patients while providing insights for healthcare practitioners for screening of depression in cancer patients and discussion of treatment options with those suffered from both conditions.

In conclusion, social-demographic characteristics and access to healthcare are determinants for pharmacotherapy of depression in US cancer patients. Policy makers and healthcare practitioners should be aware of this disparity and related barriers, and work together to find a solution to eliminate preventable inequalities and improve the health of every patient.

## Data availability statement

Publicly available datasets were analyzed in this study. This data can be found here: https://www.cdc.gov/nchs/nhanes/index.htm.

## Ethics statement

The studies involving human participants were reviewed and approved by the NCHS Ethics Review Board for NHANES protocol. The present study used publicly available de-identified data from NHANES and was exempt from IRB review. The participants provided their written informed consent to participate in the NHANES.

## Author contributions

All authors listed have made a substantial, direct, and intellectual contribution to the work and approved it for publication.

## Funding

This study was supported by Tongji Hospital, Tongji Medical College, Huazhong University of Science and Technology.

## Conflict of interest

Author YZ was employed by the company Wayzek Science. The remaining author declares that the research was conducted in the absence of any commercial or financial relationships that could be construed as a potential conflict of interest.

## Publisher's note

All claims expressed in this article are solely those of the authors and do not necessarily represent those of their affiliated organizations, or those of the publisher, the editors and the reviewers. Any product that may be evaluated in this article, or claim that may be made by its manufacturer, is not guaranteed or endorsed by the publisher.
