# Supplementary material for: Antidepressant use pattern and disparities among cancer patients in the United States
Source: Front Public Health. 2022 Nov 9;10:1000000. doi: 10.3389/fpubh.2022.1000000 (PMC9682280; doi:10.3389/fpubh.2022.1000000)
Supplement: Supplementary file 1 [file Data_Sheet_1.docx]

**Supplementary Table 1.** List of antidepressants and their classifications

| **The third level code/Drug classification** | **Medication name** |
| --- | --- |
| 250 - Monoamine oxidase inhibitors | Isocarboxazid  Phenelzine  Tranylcypromine  Selegiline  Monoamine oxidase inhibitors, unspecified |
| 306 - Phenylpiperazines | Trazodone  Nefazodone |
| 308 - Serotonin and norepinephrine reupdate inhibitors | Venlafaxine  Duloxetine  Milnacipran  Desvenlafaxine  Levomilnacipran |
| 208 - Selective serotonin reuptake inhibitors | Fluoxetine  Sertraline  Paroxetine  Fluvoxamine  Citalopram  Escitalopram |
| 209 - Tricyclic antidepressants | Serotonin  Nortriptyline  Desipramine  Amitriptyline  Doxepin  Imipramine  Trimipramine  Amoxapine  Protriptyline  Clomipramine |
| 307 - Tetracyclic antidepressants | Maprotiline  Mitrazapine |
| 76 - Miscellaneous antidepressants | Bupropion  Vilazodone  Vortioxetine  Agomelatine |

Classification of antidepressants were obtained from third level codes under the second level code of 249 for antidepressants in the 3-level nested therapeutic classification system using Lexicon Plus, a database containing information of all prescription drug products in the US. The full list of 3-level classifications and coding information for medications in NHANES can be found at https://wwwn.cdc.gov/Nchs/Nhanes/1999-2000/RXQ_DRUG.htm

**Supplementary Table 2.** Social-demographic characteristics and access to healthcare by presence of depression based on PHQ-9 scores (with 8 points as the cut-off value) among adult cancer survivors in the United States who were using antidepressants.

|  | Those with PHQ-9 score ≥8  (N=279)  Weighted N=3420732 | Those with PHQ-9 score <8  (N=143)  Weighted N=1502585 | Chi-square test  P value |
| --- | --- | --- | --- |
| Age (years) |  |  | <0.001 |
| 20-40 | 11 (110716, 3.2%) | 11 (113425, 7.5%) |  |
| 40-65 | 91 (1469421, 43.0%) | 79 (974933, 64.9%) |  |
| 65-79 | 117 (1350030, 39.5%) | 36 (306436, 20.4%) |  |
| ≥80 | 60 (490565, 14.3%) | 17 (107792, 7.2%) |  |
| Gender |  |  | 0.093 |
| Male | 98 (1123084, 32.8%) | 34 (338153, 22.5%) |  |
| Female | 181 (2297648, 67.2%) | 109 (1164432, 77.5%) |  |
| Race/Ethnicity |  |  | 0.094 |
| Non-Hispanic White | 218 (3119189, 91.2%) | 93 (1249588, 83.2%) |  |
| Non-Hispanic Black | 23 (72918, 2.1%) | 19 (77517, 5.2%) |  |
| Hispanics | 25 (93042, 2.7%) | 17 (57455, 3.8%) |  |
| Asian | 4 (14226, 0.4%) | 2 (9396, 0.6%) |  |
| Other, including multi-racial | 9 (121357, 3.5%) | 12 (108629, 7.2%) |  |
| Ratio of family income to poverty* |  |  | <0.001 |
| ≤1.85 | 95 (686258, 22.0%) | 75 (650404, 47.6%) |  |
| 1.86-3.49 | 79 (959463, 30.8%) | 28 (371252, 27.2%) |  |
| ≥3.50 | 81 (1468349, 47.2%) | 23 (345129, 25.3%) |  |
| Marital status |  |  | 0.005 |
| Married/living with partner | 162 (2308066, 67.5%) | 57 (738741, 45.6%) |  |
| Widowed/divorced/separated | 101 (939957, 27.5%) | 65 (551135, 44.2%) |  |
| Never married | 16 (172709, 5.0%) | 21 (212710, 10.3%) |  |
| Highest Education |  |  | 0.004 |
| High school diploma or lower | 97 (893592, 26.1%) | 61 (620210, 41.3%) |  |
| Associate degree | 109 (1253586, 36.6%) | 56 (611943, 40.7%) |  |
| College degree or higher | 73 (1273554, 37.2%) | 26 (270433, 18.0%) |  |
| Covered by health insurance |  |  | <0.001 |
| Yes | 275 (3368933, 98.5%) | 133 (1327114, 88.3%) |  |
| No | 4 (51799, 1.5%) | 10 (175471, 11.7%) |  |
| Routine place to go for healthcare |  |  | 0.63 |
| Yes | 273 (3329157, 97.3%) | 138 (1439601, 95.8%) |  |
| No | 6 (91575, 2.7%) | 5 (62985, 4.2%) |  |

Data were presented as sample size (weighted sample size, weighted percentage).

*Counts for this variable do not add to total number due to missing values.

**Supplementary Figure 1.** Prevalence of depression in adult cancer survivors in United States by NHANES data cycles.


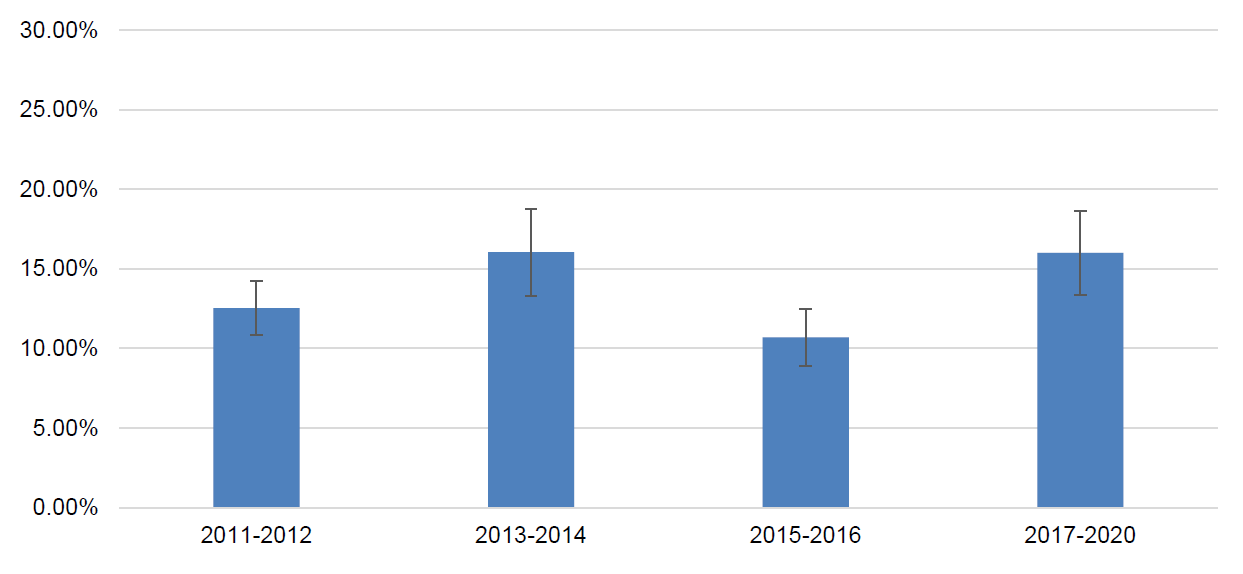


Depression was defined as the total score from PHQ-9 ≥8.
